# Long-COVID in patients with a history of mild or asymptomatic SARS-CoV-2 infection: a Nationwide Cohort Study

**DOI:** 10.1080/02813432.2022.2139480

**Published:** 2022-10-31

**Authors:** Limor Adler, Sivan Gazit, Yuval Pinto, Galit Perez, Miri Mizrahi Reuveni, Ilan Yehoshua, Robert Hoffman, Joseph Azuri, Tal Patalon

**Affiliations:** a Health Division, Maccabi Healthcare Services, Tel Aviv, Israel; b Department of Family Medicine, Sackler Faculty of Medicine, Tel Aviv University, Tel Aviv, Israel; c Kahn Sagol Maccabi (KSM) Research & Innovation Center, Maccabi Healthcare Services, Tel Aviv, Israel; d Maccabitech Institute for Research and Innovation, Maccabi Healthcare Services, Tel Aviv, Israel; e Family Medicine Residency Program, Meritus Medical Center, Hagerstwon, MD, USA; f Department of Family Medicine, Ben-Gurion University of the Negev, Beer Sheva, Israel

**Keywords:** Long-COVID, SARS-CoV-2 infection, mild disease, asymptomatic disease, cohort studies

## Abstract

**Objective:**

Evaluating the prevalence of long-COVID symptoms in patients with a history of mild or asymptomatic infection with Severe Acute Respiratory Syndrome Coronavirus 2 (SARS-CoV-2) and the factors associated with developing long-COVID.

**Design:**

A nationwide cohort study. Using a centralized database, we have identified patients with and without a history of SARS-CoV-2 infection 1–6 months before data collection. Patients were asked to fill out an online questionnaire through text messages.

**Setting:**

Israeli general practice.

**Subjects:**

2755 persons participated in the study in September 2021 (a response rate of 7.5%): 819 with and, 936 without a history of SARS-CoV-2 infection.

**Main outcome measures:**

We asked patients to provide details about their demographic status, medical history, COVID-related variables and the presence of long-COVID symptoms.

**Results:**

Most prevalent long-COVID symptoms were decreased smell sensation (35.1% *vs.* 4.3%, *p* < 0.001), decreased taste sensation (25.2% *vs.* 3.2%, *p* < 0.001), memory disturbances (36.9% *vs.* 14.4%, *p* < 0.001), dyspnea (24.2% *vs.* 10.7%, *p* < 0.001) and arthralgia (33% *vs.* 16.3%, *p* < 0.001). Risk factors associated with long-COVID included female gender, symptomatic COVID-19, overweight or obesity and the presence of dyslipidemia. About 34.6% of participants reported not returning to their baseline health condition after the acute illness.

**Conclusion:**

Long-COVID is frequently seen following a mild symptomatic COVID-19 infection and, to a lesser extent, following an asymptomatic SARS-CoV-2 infection. Primary care physicians should be aware of these symptoms and consider this option in their differential diagnosis. Health policymakers should expect a significant impact of this syndrome on public health.

**Key Points**
Long-COVID has emerged as a significant health problem with a serious impact on normal daily function
• Long-COVID symptoms were evident in patients with mild symptomatic disease and in asymptomatic patients to a lesser extent.
• Risk factors for having Long-COVID symptoms include female gender, symptomatic disease, increased BMI, and the presence of dyslipidemia.
• Fatigue, dyspnea, weakness, decreased libido, weight changes, memory, and sleep disturbances were associated with not returning to the baseline health state.

## Introduction

Severe Acute Respiratory Syndrome Coronavirus 2 (SARS-CoV-2) originated in Wuhan, China, in December 2019 and swept across the globe, impacting medical, economic and social lives. The clinical presentation of acute COVID-19 varies from asymptomatic to severe illness requiring hospitalization and intensive therapy, including non-invasive interventions and invasive ventilation. Acute symptoms include dyspnea, cough, fever, fatigue and other symptoms and often require supportive treatment [1]. Its pathophysiology is related to virus-specific pathophysiologic variations, oxidative stress, immunologic abnormalities and inflammatory damage [2,3]. COVID-19 sequelae, recognized as long-COVID or post-acute sequelae of COVID-19, is defined as symptoms lasting >4 weeks following acute infection. It is found both among patients hospitalized with severe symptoms and those who were asymptomatic or presented only mild symptoms [4]. Long-COVID’s most common symptoms include myalgia, fatigue, dyspnea, headache, joint stiffness, cough, insomnia, mood disturbances and anxiety [5,6]. Symptoms severity was not associated with the severity of the acute illness or the presence of other comorbidities [5]. Several studies aimed to characterize the clinical profiles and duration of symptoms of adults recovering from COVID-19 [5–7]. The frequency of long-COVID ranges from 4.7% to 80% [8–10]. Logue et al. followed up patients for up to 9 months after the onset of COVID-19 symptoms and reported that almost a third did not return to their baseline condition [11]. Mendelson et al. described the evolving clinical challenges regarding long-COVID [12]. While most studies focused on hospitalized patients, little is known about adults diagnosed with COVID-19 in the outpatient setting. Tenforde et al. used questionnaires to understand the resolution process of COVID-19 symptoms further and created a profile for recovering adults diagnosed in the outpatient setting [13]. 35% of the patients who participated in their study reported not returning to their usual health at the interview, describing fatigue, cough and headache as the leading clinical symptoms. In a UK-wide survey conducted through an online post-COVID-19 support and information hub, patients reported physical and psychological symptoms that fluctuate unpredictably [5]. Active smokers and females were associated with a higher risk of developing long COVID symptoms [7].

This study aimed to evaluate the prevalence of long-COVID symptoms in patients with a history of mild or asymptomatic infection with SARS-CoV-2 and to evaluate the factors associated with long-COVID and the prolonged disruption of baseline (pre-SARS-CoV-2) health status.

## Material and methods

### Study design and setting

We designed a nationwide cohort study using the centralized database of Maccabi Healthcare Services (MHS), the second-largest healthcare maintenance organization in Israel, which covers 26% of the population and provides a representative sample of the Israeli population. We identified two groups of members: those with a positive polymerase chain reaction (PCR) test for SARS-CoV-2 1–6 months before data collection and those without a positive PCR up to data collection, or SARS-CoV-2 naïve individuals (with a ratio of 1:2). We sent text messages to all patients requesting them to complete an online questionnaire (the English translation of the questionnaire is in the supplementary material). Informed consent was given *via* the online questionnaire sent in three languages, based on the patient’s preferred language, as recorded in the electronic medical record (Hebrew, Arabic and Russian). The MHS Institutional Review Board (IRB) approved this study (ID 0169-20-MHS).

### Variables

Demographic and medical variables included age, sex, smoking status and co-morbidities (hypertension, diabetes mellitus [DM], chronic obstructive pulmonary disease [COPD] or asthma, and coronary heart disease [CHD]). COVID-19-related variables included details about the acute event (if one occurred): the date of illness, presence of symptoms (without specifically mentioning which symptoms), whether the participant was admitted to a hospital due to COVID-19, and whether oxygen supply was warranted. The last section included questions regarding the presence of typical Long-COVID symptoms, each one with three possible choices (yes, no or sometimes). The questionnaire was created by the authors of this study, with relation to most symptoms reported in the literature.

### Sample size

The sample size was calculated to be 699 individuals with a history of SARS-Cov-2 infection and 1398 SARS-CoV-2 naïve individuals, based on a difference of at least 5% in prevalence of long-COVID symptoms (20% in the first group and 15% in the second group) with a significance level of 5%, 80% power, and 1:2 ratio between the two groups.

### Statistical analysis

Descriptive statistics were used for all variables, with absolute numbers and percentages for categorical variables and mean and standard deviation for continuous variables. We carried out two sets of comparisons based on the data we collected: (1) long-COVID symptoms in SARS-CoV-2 naïve individuals compared to those with a history of SARS-Cov-2 infection (with and without symptoms during the acute phase) and (2) comparing participants with a reported symptomatic SARS-CoV-2 infection compared to asymptomatic SARS-CoV-2 infection. For these analyses, we used the chi-square test. We reported both the chi-square test and the attributable risk percentage (AR[%]) for each symptom.

Additionally, we performed a multivariate logistic regression analysis of two primary outcomes in patients with a history of SARS-CoV-2 infection; the presence of any long-COVID symptoms and return to a baseline state of health. We entered age, sex and smoking status using the ENTER method and all symptoms and medical variables using the FORWARD method. We used the Statistical Package for Social Sciences (SPSS) software version 27 (SPSS Inc., Chicago, IL) for data analysis. We used WinPepi for sample size calculations.

## Results

### Study population and overall symptom frequency

In September 2021, we sent the online questionnaire to 36,744 patients (12,401 patients with a history of SARS-CoV-2 infection). 2755 (7.5%) patients consented to participate in the study and completed the online questionnaire, of which 819 with a history of SARS-CoV-2 infection and 1936 without a history of SARS-CoV-2 infection (Table 1). Of all patients with a history of SARS-CoV-2 infection, 714 (87.2%) reported they had symptomatic COVID-19, 43 reported they were hospitalized due to COVID-19 (5.3%), and 30 reported they needed oxygen supply (3.7%). The mean duration of time between confirmed infection and answering the questionnaire was 5.16 months. Overall, 694 patients (84.7%) and 1501 (77.5%) patients with and without a history of infection reported at least one symptom from our questionnaire (*p* < 0.001). 283 (34.6%). Furthermore, 385 (20.4%) of patients with and without a history of infection reported not returning to their usual health since the acute illness (*p* < 0.001) (Table 1).

**Table 1.** Characteristics of patients with and without a history of SARS-CoV-2 infection.

	Patients with a history of SARS-CoV-2 infection	Patients without a history of SARS-CoV-2 infection
	*N* = 819	*N* = 1936
Age, mean ± standard deviation	46.9 ± 14.5	47.8 ± 16.5
Age range	18–83	18–97
	*N* (%)	*N* (%)
Female	492 (60.1)	1110 (57.3)
BMI group		
<18.5	42 (5.5)	99 ( 5.9)
18.5–24.99	275 (36.3)	677 (40.1)
25–29.9	266 (35.1)	539 (31.9)
>30	156 (20.6)	321 (19)
>40	19 (2.5)	52 (3.1)
Language		
Hebrew	513 (62.6)	1608 (83.1)
Russian	282 (34.4)	304 (15.7)
Arabic	24 (2.9)	24 (1.2)
Essential Hypertension	124 (28.5)	326 (38.9)
Diabetes mellitus type 2	54 (6.8)	172 (9)
Dyslipidemia	101 (12.7)	299 (15.6)
Asthma/COPD	26 (3.3)	78 (4.1)
Ischemic heart disease	36 (4.5)	95 (5)
Oncologic disease	3 (0.4)	21 (1.1)
Smoking	125 (15.4)	369 (19.1)
Symptomatic COVID	714 (87.2)	
Presence of any symptom from our questionnaire	694 (84.7)	1501 (77.5)
Presence of at least five symptoms	474 (57.9)	715 (36.9)
Not returning to baseline state of health	283 (34.9)	385 (20.4)

### Symptoms of long-COVID

#### Comparing symptoms in participants with and without a history of SARS-CoV-2 infection

**Figure 1.**
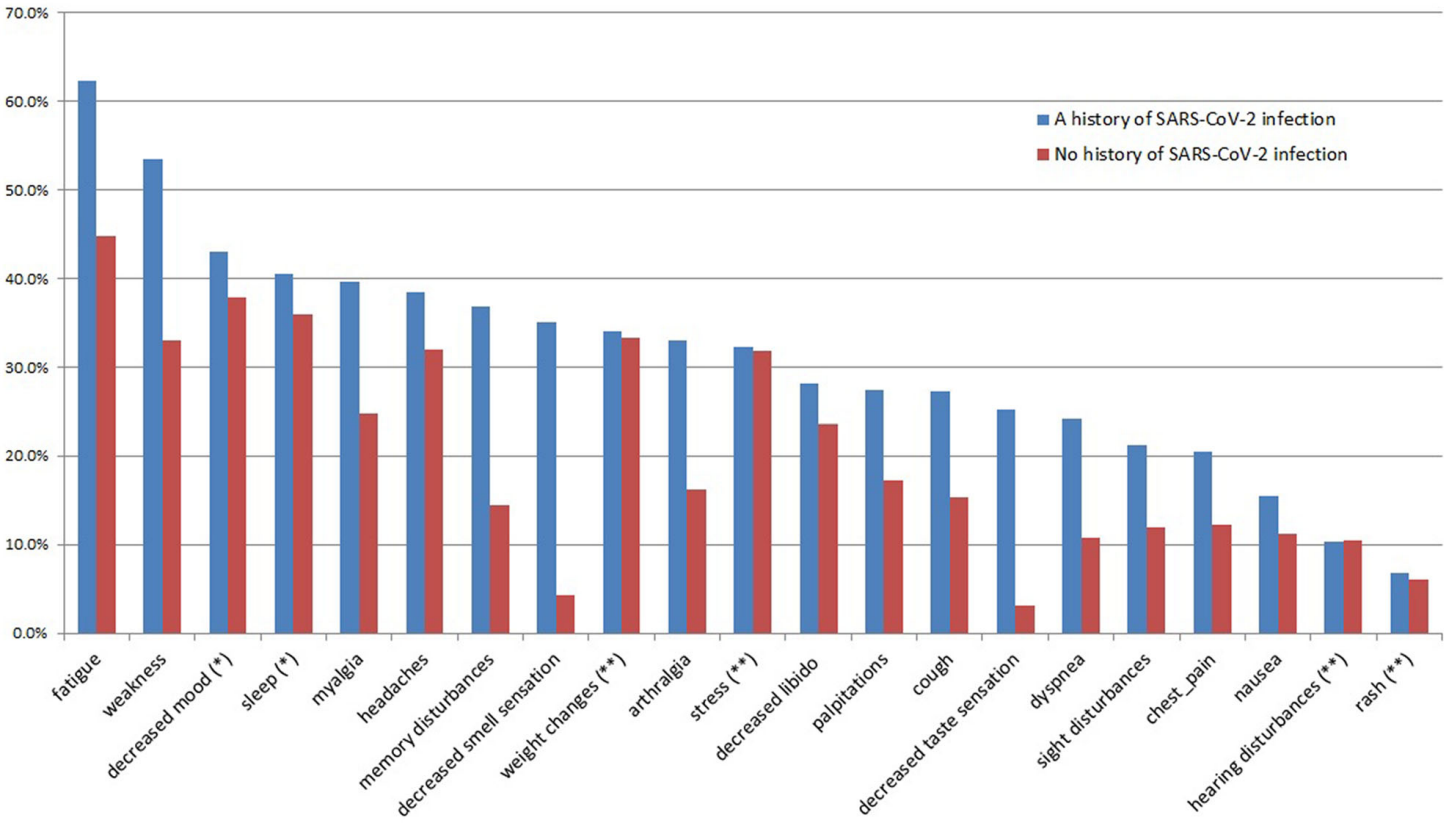
Long-COVID symptoms of patients with and without a history of SARS-CoV-2 infection.

Patients with a history of SARS-CoV-2 infection (symptomatic or asymptomatic) had more long-COVID symptoms compared to patients without such a history. Symptoms were decreased smell sensation (35.1% *vs.* 4.3%, *p* < 0.001, AR(%)=87.7%), decreased taste sensation (25.2% *vs.* 3.2%, *p* < 0.001, AR(%)=87.3%), memory disturbances (36.9% *vs.* 14.4%, *p* < 0.001, AR(%)=61%), dyspnea (24.2% *vs.* 10.7%, *p* < 0.001, AR(%)=55.4%), arthralgia (33% *vs.* 16.3%, *p* < 0.001, AR(%)=50.6%), cough (27.3% *vs.* 15.3%, *p* < 0.001, AR(%)=43.9%), disturbed vision (21.3% *vs.* 12%, *p* < 0.001, AR(%)=43.7%), chest pain (20.4% *vs.* 12.2%, *p* < 0.001, AR(%)=40.2%), weakness (53.5% *vs.* 32.9%, *p* < 0.001, AR(%)=38.5%), myalgia (39.7% *vs.* 24.7%, *p* < 0.001, AR(%)=37.8%), increased heart rate (27.5% 17.3%, *p* < 0.001, AR(%)=37.1%), fatigue (62.3% *vs.* 44.8%, *p* < 0.001, AR(%)=28%), nausea (15.4% *vs.* 11.3%, *p* = 0.003, AR(%)=26.6%), headache (38.5% *vs.* 31.9%, *p* = 0.001, AR(%)=16.8%) and decreased libido (28.2% *vs.* 23.5%, *p* = 0.012, AR(%)=16.4%) (Figure 1, Table 1S).

#### Comparing symptoms in participants with an asymptomatic SARS-CoV-2 infection to those with COVID-19

**Figure 2.**
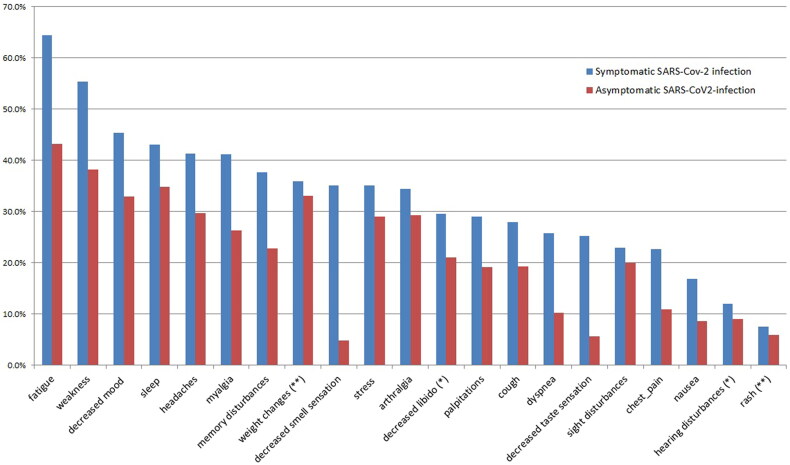
Long-COVID symptoms of patients with a symptomatic and asymptomatic history of SARS-CoV-2 infection.

Patients with COVID-19 had more long-COVID symptoms compared to participants with asymptomatic infection. Symptoms were chest pain (22.6% *vs.* 5.7%, *p* < 0.001, AR(%)=74.8%), nausea (17% *vs.* 4.8%, *p* = 0.001, AR(%)=71.8%), decreased smell sensation (38.6% *vs.* 11.4%, *p* < 0.001, AR(%)=70.5%), decreased taste sensation (27.7% *vs.* 8.7%, *p* < 0.001, AR(%)=68.6%), dyspnea (26.5% *vs.* 8.6%, *p* < 0.001, AR(%)=67.5%), headache (41.9% *vs*. 15.2%, *p* < 0.001, AR(%)=63.7%), disturbed vision (23% *vs.* 9.5%, *p* = 0.002, AR(%)=58.3%), increased heart rate (29.7% *vs*.12.4%, *p* <0.001, AR(%)=58.2%), decreased mood (46.3% *vs.* 20%, *p* < 0.001, AR(%)=56.7%), myalgia (42.7% *vs.* 19.2%, *p* < 0.001, AR(%)=55%), cough (29.4% *vs.* 13.3%, *p* < 0.001, AR(%)=54.8%), memory disturbances (39.7% *vs.* 18.1%, *p* < 0.001, AR(%)=54.4%), sleep disturbances (43.5% *vs.* 21%, *p* < 0.001, AR(%)=51.7%), stress (34.5% *vs.* 17.1%, *p* < 0.001, AR(%)=50.4%), fatigue (66.6% *vs.* 33.3%, *p*<.001, AR(%)=50%), arthralgia (35.2% *vs.* 18.1%, *p* < 0.001, AR(%)=48.5%) and weakness (57% *vs.* 29.5%, *p* < 0.001, AR(%)=48.2%) (Figure 2, Table 2S).

**Table 2.** Multivariate analysis of patients with a history of SARS-CoV-2 infection who reported at least one symptom of long COVID-19.

Variable	Odds ratio (95% confidence interval)	*p* Value
Age^a^	0.99 (0.98, 1.01)	0.750
Sex (female)^a^	5.1 (3.15, 8.27)	<0.001
Time from illness^a^	0.97 (0.92, 1.03)	0.283
Smoker^a^	1.64 (0.87, 3.10)	0.129
Symptomatic COVID-19^b^	4.28 (2.45, 7.48)	<0.001
Dyslipidemia^b^	2.51 (1.02, 6.20)	0.045
BMI_group^b^		
BMI < 18.5	0.72 (0.27, 1.83)	0.495
BMI 18.5–25	Reference	
BMI > 25	2.64 (1.54, 4.52)	<0.001
BMI_group > 30	3.45 (1.68, 7.09)	0.001

^a^Age, sex, time from illness, and smoking status were entered with ENTER method.

^b^Symptomatic illness, hospitalization, hypertension, diabetes mellitus type 2, dyslipidemia, chronic obstructive sleep apnea/asthma, heart disease, oncologic disease and BMI group were entered with the FORWARD method.

### Multivariate analysis

#### Risk factors associated with long-COVID

The presence of any long-COVID symptom was associated with female gender (odds ratio [OR] = 5.1, 95% CI 3.15, 8.27, *p* < 0.001), symptomatic SARS-CoV-2 infection (OR = 4.28, 95% CI 2.45, 7.48, *p* < 0.001), dyslipidemia (OR = 2.51, 95% CI 1.02, 6.20, *p* = 0.045) and increased body mass index (BMI) (OR = 2.64, 95% CI 1.54, 4.52, *p* < 0.001 and OR = 3.45, 95% CI 1.68, 7.09, *p* = 0.001 for BMI > 25 or BMI > 30, respectively) (Table 2).

#### Return to a baseline state of health

Not returning to a baseline state of health was associated with fatigue (OR = 3.34, 95% CI 1.62, 6.89, *p* < 0.001), dyspnea (OR =2.91, 95% CI 1.76, 4.81, *p* < 0.001), weakness (OR = 3.64, 95% CI 2.03, 6.50 *p* < 0.001), memory disturbances (OR = 2.18, 95% CI 1.37, 3.46, *p* = 0.001), decreased libido (OR = 2.03, 95% CI 1.25,3.30, *p* = 0.004), sleep disturbances (OR = 2.14, 95% CI 1.33, 3.47, *p* = 0.002), increased heart rate (OR = 1.75, 95% CI 1.08, 2.83, *p* = 0.023) and weight change (OR = 2.36, 95% CI 1.51, 3.70, *p*< 0.001). A history of ischemic heart disease was associated with better return to usual state of health (OR = 0.34, 95% CI 0.12, 0.95, *p* = 0.039) (Table 3).

**Table 3.** Multivariate analysis of patients with a history of SARS-CoV-2 infection who reported their overall health state is worse than before the illness.

Variable	Odds ratio (95% confidence interval)	*p* Value
Age^a^	0.99 (0.98,1.01)	0.288
Sex (female)^a^	0.69 (0.42,1.12)	0.131
Time from illness^a^	1.00 (0.95,1.06)	0.930
Smoker^a^	0.87 (0.48, 1.55)	0.630
Heart disease^b^	0.34 (0.12, 0.95)	0.039
Fatigue^b^	3.34 (1.62,6.89)	0.001
Dyspnea^b^	2.91 (1.76, 4.81)	<0.001
Weakness^b^	3.64 (2.03, 6.50)	<0.001
Memory problems^b^	2.18 (1.37, 3.46)	0.001
Decreased libido^b^	2.03 (1.25,3.30)	0.004
Sleep disturbances^b^	2.14 (1.33, 3.47)	0.002
Increased heart rate^b^	1.75 (1.08, 2.83)	0.023
Weight changes^b^	2.36 (1.51, 3.70)	<0.001

^a^Age, sex, time from illness and smoking status were entered with ENTER method.

^b^Symptomatic illness, hospitalization, hypertension, diabetes mellitus type 2, dyslipidemia, chronic obstructive sleep apnea/asthma, heart disease, oncologic disease, BMI group, and long COVID-19 s symptoms (fatigue, decreased smell sensation, decreased taste sensation, headache, dyspnea, myalgia, cough, rash, nausea, weakness, depression, stress, memory disturbances, decreased libido, sleep disturbances, arthralgia, chest pain, increased heart rate, disturbed vision, hearing disturbances) were entered with the FORWARD method.

## Discussion

### Principal findings

In this nationwide cohort study, we have found that the most prevalent long-COVID symptoms were a decreased sensation of smell and taste, memory disturbances, dyspnea, arthralgia, cough, vision disturbances, chest pain, weakness, myalgia, increased heart rate, fatigue, nausea, headache and decreased libido. When comparing individuals with COVID-19 to asymptomatic individuals with SARS-CoV-2 infection, long-COVID symptoms were the same, with stress also more prevalent in symptomatic patients.

Factors associated with at least one long-COVID symptom include female gender, symptomatic SARS-CoV-2 infection, dyslipidemia and overweight or obesity. In addition, factors associated with not returning to a baseline state of health were long-COVID symptoms, including fatigue, dyspnea, weakness, memory disturbances, decreased libido, sleep disturbances, increased heart rate and weight changes.

### Strengths and limitations

The strengths of this study are its nationwide coverage, the dissemination of the questionnaire in the three most commonly spoken languages in Israel (Hebrew, Arabic and Russian), and the focus on patients who were almost entirely treated in the community with mild or asymptomatic infection. This population is much less studied for long-COVID than hospitalized patients. In addition, the number of participants (*n* = 2755, while most studies had only a few hundreds [14]) and the comparison to healthy respondents represent other strengths. The limitations of this study are the low response rate (7.5%) and a possible selection bias due to the voluntary nature of such a questionnaire; patients who had symptoms were probably the most willing to report them. We did not ask open questions or ask about the severity of the symptoms reported. In addition, no follow-up was done to examine whether alleviation of symptoms occurred.

The low response rate is reasonable due to the study design; most studies on long-COVID were conducted through outpatients’ visits after recovery or phone interviews, and only a minority through an electronic survey. The response rate to interview during an outpatient visit or even during a phone interview is expected to be higher than in electronic surveys. Nevertheless, this approach allows us to reach out to patients who otherwise would probably not participate in such studies because they had a mild or asymptomatic illness.

### Findings in relation to other studies

#### Symptoms of long-COVID

Long-COVID has a significant impact on public health, especially in primary care medicine [15]. Therefore, in March 2021, the World Health Organization recommended emphasizing the considerable impact of long-COVID on people’s ability to function normally (work or social life) [16].

The frequency of at least one symptom reported from the questionnaire was 84.7% in patients with and 77.5% in patients without a history of SARS-CoV-2 infection (*p* < 0.001). This finding suggests that these symptoms are frequently seen in all patients, and although the difference is significant, it is worth mentioning. The frequency of long-COVID in other studies ranged from 4.7% to 80% [8,10].

Our results are in-line with other studies that revealed similar long-COVID symptoms; most reviews and meta-analyses showed weakness, general malaise, fatigue, dyspnea (also referred to as breathlessness), arthralgia and headache were the most prevalent symptoms [10,14,17,18]. Other symptoms include concentration impairment, cough, chest pain or tightness, loss of smell and taste, myalgia, sore throat, diarrhea, memory disturbances, depression, anxiety and sleep disturbances [18–22]. Cardiovascular symptoms included arrhythmias, palpitations and hypotension, increased heart rate, venous thromboembolic diseases, myocarditis and acute heart failure [18].

In a systemic review that focused on patients with mild disease, the prevalence of long-COVID symptoms was 10–35%, with fatigue as the most frequent symptom. Other symptoms were dyspnea, cough, chest pain, headache, decreased mental and cognitive status and loss of the sensation of smell [23]. In another study on patients with mild disease, the most prevalent long-COVID symptoms were cough, fatigue and shortness of breath [13].

Interestingly, in our study, all long-COVID symptoms were reported to some extent in healthy individuals. Lockdown and social distancing due to the pandemic influenced the general population, even those who were not infected; most research focused on mental help and showed an increase in the prevalence of depression, anxiety and sleep disturbances during the pandemic [24]. However, these complaints may often be found in the general population in times of sickness or even in health. This should raise questions regarding whether they genuinely represent long-COVID symptoms or merely malaise complaints that may be found in all patients from time to time. Another possibility is that long-COVID symptoms are related to a long recovery time that exists in many viral infections. Hickie et al. showed that 12% of patients recovering from viral infections may exhibit disabling fatigue and musculoskeletal pain mood disturbances [25].

#### Risk factors associated with long-COVID

In our study, factors associated with the development of long-COVID included female gender, symptomatic COVID-19, overweight (BMI > 25) or obesity (BMI > 30), and the presence of dyslipidemia. These results align with other studies that showed an association between the female gender and symptomatic illness and long-COVID [7,26,27]. We did not find an association between hospital admission and oxygen supplementation at the acute phase with long-COVID, although other studies found this association [8].

#### Return to a baseline state of health

In our study, 283 (34.6%) participants reported not returning to their baseline health after the acute illness. This is in line with other studies that showed that more than a third of patients reported reduced quality of life [14]. In addition, patients with mild disease had suffered long-COVID symptoms with significant consequences on work and daily functioning [23].

We found that most factors associated with not returning to the patient’s usual state of health were long-COVID symptoms, including fatigue, dyspnea, weakness, memory disturbances, decreased libido, sleep disturbances, increased heart rate and weight changes. Obesity was not associated with not returning to one’s usual health, although a different study did find such an association [13].

#### Meaning of the study

The impact of COVID-19 on human health is immense and evolving. This is a dynamic and constantly changing field of research and health care, with unpredictable consequences. One area of concern is the long-term health impact of SARS-CoV-2 infection. Long-COVID may significantly impact human health in years to come, and surveys to date estimate that 80% of patients will develop at least one symptom following SARS-CoV-2 infection [10]. Our study found that 34.6% of patients report not returning to their previous state of health (with a mean duration of 5 months after the SARS-CoV-2 infection).

Primary healthcare practitioners and healthcare providers can expect long-COVID to be a significant source of healthcare demands in the coming months and perhaps years.

It is concerning to find that long-COVID is frequently seen following a mild COVID-19 infection and, to a lesser extent, following asymptomatic SARS-CoV-2 infection. Primary care physicians should be aware of these symptoms and consider this option in their differential diagnosis. Further research is needed to develop and evaluate treatment options for these patients.

## Supplementary Material

Supplemental Material
