# Supplementary material for: Long-COVID in patients with a history of mild or asymptomatic SARS-CoV-2 infection: a Nationwide Cohort Study
Source: Scand J Prim Health Care. 2022 Oct 31;40(3):342–9. doi: 10.1080/02813432.2022.2139480 (PMC9848375; doi:10.1080/02813432.2022.2139480)
Supplement: Supplemental Material [file IPRI_A_2139480_SM3246.docx]

# Supplementary materials

Table 1S. Univariate comparison of symptoms of patients with and without a history of SARS-CoV-2 infection

| **Symptom** | **No history of SARS-CoV2-infection** | **With a history of SARS-Cov-2 infection** | **Relative risk* (95% confidence interval)** | **Attributable Risk (%)*** | **P value** |
| --- | --- | --- | --- | --- | --- |
|  | **N=1936**  **n (%)** | **N=819**  **n (%)** |  |  |  |
| Relative risk > 2 | | | | | |
| **Decreased smell sensation**  No  Sometimes  yes | 1819 (95.7)  36 (1.9)  45 (2.4) | 529 (64.9)  88 (10.8)  198 (24.3) | 8.23  (6.52,10.39) | 87.7 | <0.001 |
| **Decreased taste sensation**  No  Sometimes  yes | 1831 (96.8)  23 (1.2)  37 (2.0) | 601 (74.8)  63 (7.8)  140 (17.4) | 7.96 (6.04,10.49) | 87.3 | <0.001 |
| **Memory disturbances**  No  Sometimes  yes | 1439 (85.6)  64 (3.8)  178 (10.6) | 515 (63.1)  134 (16.4)  167 (20.5) | 2.56 (2.21,2.97) | 61.0 | <0.001 |
| **Dyspnea**  No  Sometimes  yes | 1697 (89.3)  140 (7.4)  64 (3.4) | 618 (75.8)  122 (15)  75 (9.2) | 2.25 (1.89,2.69) | 55.4 | <0.001 |
| **Arthralgia**  No  Sometimes  yes | 1445 (83.7)  77 (4.5)  204 (11.8) | 547 (67)  116 (14.2)  153 (18.8) | 2.02 (1.75,2.34) | 50.6 | <0.001 |
| Relative risk > 1 | | | | | |
| **Cough**  No  Sometimes  yes | 1608 (84.7)  205 (10.8)  85 (4.5) | 593 (72.7)  125 (15.3)  98 (12) | 1.79 (1.53,2.09) | 43.9 | <0.001 |
| **Visual disturbance**  No  Sometimes  yes | 1577 (88)  162 (9)  53 (3) | 641 (78.7)  74 (9.1)  99 (12.2) | 1.77 (1.48,2.13) | 43.7 | <0.001 |
| **Chest pain**  No  Sometimes  yes | 1662 (87.8)  155 (8.2)  76 (4) | 647 (79.6)  83 (10.2)  83 (10.2) | 1.67 (1.39,2.01) | 40.2 | <0.001 |
| **Weakness**  No  Sometimes  yes | 1274 (67.1)  378 (19.9)  248 (13.1) | 380 (46.5)  190 (23.3)  247 (30.2) | 1.62 (1.48,1.78) | 38.5 | <0.001 |
| **Myalgia**  No  Sometimes  yes | 1430 (75.3)  274 (14.4)  196 (10.3) | 492 (60.3)  151 (18.5)  173 (21.2) | 1.60 (1.43,1.80) | 37.8 | <0.001 |
| **Increased heart rate**  No  Sometimes  yes | 1570 (82.7)  228 (12)  100 (5.3) | 591 (72.5)  120 (14.7)  104 (12.8) | 1.59 (1.37,1.84) | 37.1 | <0.001 |
| **Fatigue**  No  Sometimes  yes | 1050 (55.2)  526 (27.7)  326 (17.1) | 308 (37.7)  219 (26.8)  291 (35.6) | 1.39 (1.29,1.50) | 28.0 | <0.001 |
| **Nausea**  No  Sometimes  yes | 1684 (88.7)  135 (7.1)  79 (4.2) | 691 (84.6)  68 (8.3)  58 (7.1) | 1.37 (1.11,1.68) | 26.6 | 0.003 |
| **Headaches**  No  Sometimes  yes | 1291 (68.1)  373 (19.7)  233 (12.3) | 502 (61.5)  150 (18.4)  164 (20.1) | 1.20 (1.08,1.34) | 16.8 | 0.001 |
| **Decreased libido**  No  Sometimes  yes | 1421 (76.5)  236 (12.7)  201 (10.8) | 569 (71.8)  115 (14.5)  108 (13.6) | 1.20 (1.04,1.37) | 16.4 | 0.012 |
| The sample size is not big enough to prove an association | | | | | |
| **Decreased mood**  No  Sometimes  yes | 1180 (62.1)  399 (21)  320 (16.9) | 466 (57)  173 (21.2)  178 (21.8) | 1.13 (1.03,1.25) |  | 0.013 |
| **Sleep disturbance**  No  Sometimes  yes | 1216 (64.1)  378 (19.9)  303 (16) | 483 (59.4)  152 (18.7)  178 (21.9) | 1.13 (1.02,1.25) |  | 0.022 |
| Non-significant results | | | | | |
| **Rash**  No  Sometimes  yes | 1783 (93.9)  61 (3.2)  54 (2.8) | 760 (93.1)  18 (2.2)  38 (4.7) | 1.13 (0.83,1.54) |  | 0.439 |
| **Stress or increased worries**  No  Sometimes  yes | 1298 (68.2)  373 (19.6)  232 (12.2) | 552 (67.7)  144 (17.7)  119 (14.6) | 1.01 (0.90,1.14) |  | 0.822 |
| **Hearing disturbances**  No  Sometimes  yes | 1693 (89.6)  103 (5.4)  94 (5) | 713 (89.7)  48 (6)  34 (4.3) | 0.99 (0.77,1.26) |  | 0.945 |
| **Weight changes**  No  yes | 1264 (66.7)  631 (33.3) | 553 (67.9)  262 (32.1) | 1.02 (0.91,1.15) |  | 0.723 |

*relative risks and attributable risks were calculated based on “yes” and “sometimes” combined to one variable and compared to “no” (not having the symptom at all).

Table 2S. Univariate comparison of symptoms of patients with a symptomatic and asymptomatic history of SARS-CoV-2 infection

| **Symptom** | **Asymptomatic SARS-CoV2-infection** | **Symptomatic SARS-Cov-2 infection** | **Relative risk* (95% confidence interval)** | **Attributable Risk (%)*** | **P value** |
| --- | --- | --- | --- | --- | --- |
|  | **N=105**  **n (%)** | **N=714**  **n (%)** |  |  |  |
| Relative risk > 2 | | | | | |
| **Chest pain**  No  Sometimes  yes | 99 (94.3)  4 (3.8)  2 (1.9) | 548 (77.4)  79 (11.2)  81 (11.4) | 3.95 (1.80, 8.70) | 74.8 | <0.001 |
| **Nausea**  No  Sometimes  yes | 100 (95.2)  4 (3.8)  1 (1) | 591 (83)  64 (9)  57 (8) | 3.57 (1.49, 8.52) | 71.8 | 0.001 |
| **Decreased smell sensation**  No  Sometimes  yes | 93 (88.6)  5 (4.8)  7 (6.7) | 436 (61.4)  83 (11.7)  191 (26.9) | 3.38 (1.97, 5.80) | 70.5 | <0.001 |
| **Decreased taste sensation**  No  Sometimes  yes | 95 (91.3)  3 (2.9)  6 (5.8) | 506 (72.3)  60 (8.6)  134 (19.1) | 3.20 (1.70,6.05) | 68.6 | <0.001 |
| **Dyspnea**  No  Sometimes  yes | 96 (91.4)  8 (7.6)  1 (1) | 522 (73.5)  114 (16.1)  74 (10.4) | 3.09 (1.63, 5.84) | 67.5 | <0.001 |
| **Headaches**  No  Sometimes  yes | 89 (84.8)  11 (10.5)  5 (4.8) | 413 (58.1)  139 (19.5)  159 (22.4) | 2.75 (1.74, 4.35) | 63.7 | <0.001 |
| **Disturbed vision**  No  Sometimes  yes | 95 (90.5)  5 (4.8)  5 (4.8) | 546 (77)  69 (9.7)  94 (13.3) | 2.41 (1.32, 4.42) | 58.3 | 0.002 |
| **Increased heart rate**  No  Sometimes  yes | 92 (87.6)  7 (6.7)  6 (5.7) | 499 (70.3)  113 (15.9)  98 (13.8) | 2.40 (1.42, 4.04) | 58.2 | <0.001 |
| **Decreased mood**  No  Sometimes  yes | 84 (80)  13 (12.4)  8 (7.6) | 382 (53.7)  160 (22.5)  170 (23.9) | 2.32 (1.57, 3.42) | 56.7 | <0.001 |
| **Myalgia**  No  Sometimes  yes | 84 (80.8)  18 (17.3)  2 (1.9) | 408 (57.3)  133 (18.7)  171 (24) | 2.22 (1.48, 3.32) | 55.0 | <0.001 |
| **Cough**  No  Sometimes  yes | 91 (86.7)  7 (6.7)  7 (6.7) | 502 (70.6)  118 (16.6)  91 (12.8) | 2.20 (1.34, 3.64) | 54.8 | <0.001 |
| **Memory disturbances**  No  Sometimes  yes | 86 (81.9)  9 (8.6)  10 (9.5) | 429 (60.3)  125 (17.6)  157 (22.1) | 2.19 (1.44, 3.33) | 54.4 | <0.001 |
| **Sleep disturbance**  No  Sometimes  yes | 83 (79)  9 (8.6)  13 (12.4) | 400 (56.5)  143 (20.2)  165 (23.3) | 2.08 (1.42, 3.04) | 51.7 | <0.001 |
| **Stress or increased worries**  No  Sometimes  yes | 87 (82.9)  10 (9.5)  8 (7.6) | 465 (65.5)  134 (18.9)  111 (15.6) | 2.01 (1.31, 3.10) | 50.4 | <0.001 |
| **Fatigue**  No  Sometimes  yes | 70 (66.7)  26 (24.8)  9 (8.6) | 238 (33.4)  193 (27.1)  282 (39.5) | 2.00 (1.52, 2.63) | 50.0 | <0.001 |
| Relative risk > 1 | | | | | |
| **Arthralgia**  No  Sometimes  yes | 86 (81.9)  13 (12.4)  6 (5.7) | 461 (64.8)  103 (14.5)  147 (20.7) | 1.94 (1.28, 2.95) | 48.5 | <0.001 |
| **Weakness**  No  Sometimes  yes | 74 (70.5)  25 (23.8)  6 (5.7) | 306 (43)  165 (23.2)  241 (33.8) | 1.93 (1.43, 2.61) | 48.2 | <0.001 |
| The sample size is not big enough to prove an association | | | | | |
| **Hearing disturbances**  No  Sometimes  yes | 100 (96.2)  2 (1.9)  2 (1.9) | 613 (88.7)  46 (6.7)  32 (4.6) | 2.93 (1.10, 7.85) |  | 0.02 |
| **Decreased libido**  No  Sometimes  yes | 85 (81.7)  12 (11.5)  7 (6.7) | 484 (70.3)  103 (15)  101 (14.7) | 1.62 (1.06, 2.47) |  | 0.016 |
| Non-significant results | | | | | |
| **Rash**  No  Sometimes  yes | 99 (94.3)  3 (2.9)  3 (2.9) | 661 (93)  15 (2.1)  35 (4.9) | 1.23 (0.54, 2.80) |  | 0.618 |
| **Weight changes**  No  yes | 75 (72.1)  29 (7.9) | 462 (65)  249 (35) | 1.25 (0.91,1.74) |  | 0.152 |

*relative risks and attributable risks were calculated based on “yes” and “sometimes” combined to one variable and compared to “no” (not having the symptom at all).

# The questionnaires

We hereby send you a request to take part in a study conducted by Maccabi Healthcare Services. We would like to you answer several questions. You can choose not to take part in this questionnaire or to respond to some or all of the questions. The information gathered from this questionnaire is completely confidential and will be used only for the sake of this study.

Do you agree to the participation in this study? Yes/No

Part A: for patients with a history of SARS CoV-2 infection

A.1

When did you have COVID-19 infection? (Month/Year)

During the COVID-19infection - Did you have any symptoms? Yes/No

During the COVID-19 infection were you admitted to hospital? Yes/No

If the answer is yes, were you on respiratory support or admitted to intensive care unit? Yes/No

Age? ____

Gender? Female / male

A.2

Do you feel you recovered completely from the COVID-19 infection? Yes/no.

Below is a list of symptoms.

Please mark if you suffered from any of these symptoms more often than before the COVID-19 infection? Yes/sometimes/no

- Fatigue
- Change in sense of smell
- Change in the sense of taste
- Headache
- Shortness of breath
- Muscle pains
- Cough
- Rash
- Nausea
- Weakness
- Decreased mood
- Anxiety or excessive worrying
- Memory deterioration
- A decrease in your libido
- Sleeping disturbances
- Joints aches
- Abdominal Pain
- Chest Pain
- Palpitations/Heart racing
- Vision disturbance
- Hearing disturbance
- A decrease or increase (>3 kg) in the past year
- Other symptoms? ______________

In general, how is your health in comparison to the condition before the COVID-19 infection? Same/Better/Worse

A.3

Are you diagnosed with or receive medical treatment for one of these conditions:

- Hypertension
- Diabetes mellitus
- Dyslipidemia
- Asthma or chronic lung condition
- Heart disease
- Oncologic disorder

Do you smoke? Yes/No

Were you born in Israel? Yes/No

Weight: ________ Height: ________

We appreciate your cooperation!

Part B: for patients without a history of SARS CoV-2 infection

B.1

Age? ____

Gender? Female / male

B.2

Below is a list of symptoms.

Please mark if you suffered from any of these symptoms more often than before the COVID-19 pandemic? Yes/sometimes/no

- Fatigue
- Change in sense of smell
- Change in the sense of taste
- Headache
- Shortness of breath
- Muscle pains
- Cough
- Rash
- Nausea
- Weakness
- Decreased mood
- Anxiety or excessive worrying
- Memory deterioration
- A decrease in your libido
- Sleeping disturbances
- Joints aches
- Abdominal Pain
- Chest Pain
- Palpitations/Heart racing
- Vision disturbance
- Hearing disturbance
- A decrease or increase (>3 kg) in the past year
- Other symptoms? ______________

In general, how is your health status compared to how it was before the COVID-19 pandemic? Same/Better/Worse

B.3

Are you diagnosed with or receive medical treatment for one of these conditions:

- Hypertension
- Diabetes mellitus
- Dyslipidemia
- Asthma or chronic lung condition
- Heart disease
- Oncologic disorder

Do you smoke? Yes/No

Were you born in Israel? Yes/No

Weight: ________ Height: ________

We appreciate your cooperation!
